# *CYP17A1* and Blood Pressure Reactivity to Stress in Adolescence

**DOI:** 10.1155/2015/734586

**Published:** 2015-01-26

**Authors:** Mariel Van Woudenberg, Jean Shin, Manon Bernard, Catriona Syme, Michal Abrahamowicz, Gabriel Leonard, Michel Perron, Louis Richer, Suzanna Veillette, Daniel Gaudet, Tomas Paus, Zdenka Pausova

**Affiliations:** ^1^The Hospital for Sick Children, University of Toronto, Toronto, ON, Canada M5G 1X8; ^2^Department of Epidemiology, Biostatistics and Occupational Health, McGill University, Montreal, QC, Canada H3A 0G4; ^3^Montreal Neurological Institute and Hospital, McGill University, Montreal, QC, Canada H3A 2B4; ^4^Université du Québec à Chicoutimi, Chicoutimi, QC, Canada G7H 2B1; ^5^Community Genomic Centre, Chicoutimi Hospital, Université de Montréal, Montreal, QC, Canada G7H 5H6; ^6^Rotman Research Institute, University of Toronto, Toronto, ON, Canada M6A 2E1

## Abstract

Adolescents who exhibit exaggerated blood pressure (BP) reactivity to physical and mental challenges are at increased risk of developing hypertension in adulthood. BP at rest and in response to challenges is higher in males than females, beginning in early adolescence. *CYP17A1* is one of the well-established gene loci of adult hypertension. Here, we investigated whether this gene locus is associated with elevated BP at rest and in response to physical (active standing) and mental (math stress) challenges in adolescence. We studied 496 male and 532 female adolescents (age 12–18 years) who were recruited from a genetic founder population. Our results showed that the variant of *CYP17A1* rs10786718 was associated with enhanced BP reactivity to the mental but not physical challenge and in males but not females. In males, BP increase in response to math stress was higher in major versus minor allele homozygotes by 7.6 mm Hg (*P* = 8.3 × 10^−6^). Resting BP was not associated with the *CYP17A1* variant in either sex. These results suggest that, in adolescent males but not females, *CYP17A1* enhances BP reactivity to mental stress. Whether this effect contributes to the higher prevalence of hypertension in males than females later in life remains to be determined.

## 1. Introduction

A growing body of evidence suggests that preclinical features of hypertension emerge already during adolescence [1–3], which is a period of human development when adult blood pressure (BP) and body composition develop [4, 5]. Enhanced BP reactivity to a physical or mental stressor in childhood and adolescence predicts adult hypertension [6–8]. Marked sex differences exist in resting BP and BP reactivity as well as in the prevalence of hypertension, with all being higher in males than females during reproductive years [6, 9–13].

**Figure 1:**
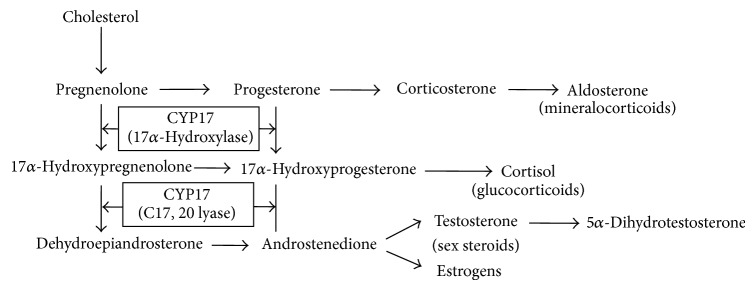
Simplified pathway of steroid hormone biosynthesis and the role of* CYP17A1*.* CYP17A1* encodes the enzyme cytochrome P-450c17*α* (CYP17) that catalyzes steroid 17*α*-hydroxylase and 17,20-lyase activities and is hence essential for the synthesis of glucocorticoids (17*α*-hydroxylase activity) and sex steroids (17,20 lyase activity). Adapted from Molina and Belldegrun, 2011 [28].

Susceptibility to hypertension is determined by both genetic and environmental factors. The estimated heritability of resting BP and hypertension is around 50% [14]. Similarly, the estimated heritability of BP response to math stress is around 50% [15]. The underlying genetic architecture involves multiple contributory genes. One of the well-established gene loci of hypertension is* CYP17A1* [16–22]. This gene encodes the enzyme cytochrome P-450c17 that mediates steroid 17*α*-hydroxylase and 17,20-lyase activities (Figure 1). The first enzymatic activity is key in the steroidogenic pathway that produces mineralocorticoids, which affect sodium and water handling in the kidney, and glucocorticoids, which control the body response to stress [23, 24]. The second enzymatic activity is involved in the biosynthesis of male and female sex hormones [24]. Thus,* CYP17A1* may influence BP reactivity in a sex-specific manner. While* CYP17A1* is associated with resting BP and hypertension in adults, whether the gene locus influences BP reactivity and whether it plays a role in BP regulation already in adolescence have not been studied.

The aim of this study was to investigate whether* CYP17A1* is associated with BP at rest and in response to physical and mental challenges during adolescence. This investigation was carried out in a community-based sample of 1,028 adolescents recruited from a genetic founder population of Quebec, Canada [25–27].

## 2. Methods and Procedures

### 2.1. Study Population

White Caucasian adolescents (496 males, 532 females), aged 12 to 18 years, were recruited from a genetic founder population living in the Saguenay-Lac St. Jean region of Quebec, Canada, as part of the Saguenay Youth Study [29]. This is a community-based cohort investigating cardiometabolic and brain health in adolescence. Male and female participants were recruited via high schools; detailed recruitment and selection criteria are described elsewhere [29]. The Saguenay Youth Study is family based, focusing on collection of sib pairs.

The Saguenay-Lac St. Jean population is one of the largest founder populations in North America [25–27], originating from French ancestors who migrated to the region in the early 19th century. The population grew from 5,200 inhabitants in 1852 to 285,000 at present, due to high intrinsic growth and little emigration. Because of the founder effect, there is a higher prevalence of certain recessive disorders in the Saguenay-Lac St. Jean region compared with other populations [25], as well as limited allelic diversity among patients with these disorders [26, 27].

The current study sample consists of 496 male and 532 female adolescents recruited and tested between November 2003 and February 2012. The prevalence of hypertension (sitting SBP or DBP ≥95th age-, sex-, and height-specific percentile) in this sample was 7.1% in males and 3.3% in females, which is similar to that in the Canadian adolescent population at large (Canadian Health Measures Survey) [30]. Additional characteristics of the studied males and females are provided in Table 1. Written consent from the parents and assent from the adolescents were obtained before the commencement of data collection. The research ethics committees of the Chicoutimi Hospital and the Hospital for Sick Children in Toronto approved the study protocols.

### 2.2. Assessments

**Figure 2:**
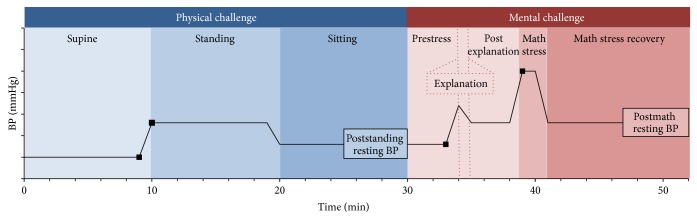
Schematic diagram of the cardiovascular protocol. The cardiovascular protocol consisted of one physical challenge and one mental challenge. The physical challenge was active posture test, which consisted of three periods during which the participant was first supine (10 minutes), then standing (10 minutes), and finally sitting (10 minutes). The mental challenge was a math-stress test, which consisted of a prestress (4 min), explanation (~1 min), postexplanation waiting (4 minutes), math-stress (2 minutes), and math-stress recovery (10 minutes) periods. Black squares indicate the initial and final BP values used to calculate BP reactivity to standing and math stress and the boxes show 5-minute averages of poststanding and postmath resting BPs.

All adolescents underwent a 52-minute cardiovascular protocol, conducted in the Chicoutimi Hospital on Saturdays, commencing between 8:00 and 12:00 [29]. The protocol consisted of physical and mental challenges (Figure 2). The physical challenges were changes in posture: each participant was first supine for 10 min, then standing for 10 min, and finally sitting for 10 min. The mental challenge was a math-stress test, consisting of an explanation (<1 min), postexplanation waiting period (4 min), math stress (2 min), and math-stress recovery (10 min). The math stress was a sequence of 46 simple arithmetic problems of increasing difficulty (to ensure some failure in all participants) to be solved out loud.

Throughout the protocol, a noninvasive hemodynamic monitor, Finometer (FNS Finapres, Amsterdam, Netherlands), was used to record continuous finger blood flow. The Finometer derives beat-by-beat brachial systolic BP (SBP) and diastolic BP (DBP) by the reconstruction and level-correction of the finger blood-flow waveform. This method has been validated for tracking BP in adults and children over the age of six years [31]. One-minute averages of these data were calculated for the duration of the protocol and used to compute two BP reactivity parameters and two resting BPs for each SBP and DBP (Figure 2). The BP reactivity parameters were (1)* BP reactivity to standing*, which was a change in BP from the last minute of supine to the first minute of standing, and (2)* BP reactivity to math stress*, which was a change in BP from the last minute of the prestress period to the first minute of the math stress. The two resting BPs were (1)* poststanding resting BP*, which was the average BP during the last 5 minutes of the 10 min sitting period during the posture test, and (2)* postmath resting BP*, which was the average BP during the last 5 min of the 10-min period following the math stress. Resting BPs were intended to mimic “clinic” BP, defined by the Canadian Hypertension Society as repeated BP measurements taken after 5 minutes of rest while seated [32]. Sex-specific means and standard deviations of all initial and resting BPs for SBP and DBP are presented in Table 1.

### 2.3. Genotyping and Genotype Imputation

The study population (*n* = 1,028) was genotyped in two waves. The first wave, involving 601 participants of the SYS, was genotyped with the Illumina Human610-Quad BeadChip (Illumina, San Diego, CA; *n* = 582,892 SNPs) at the Centre National de Génotypage (Paris, France). The second wave, involving the remaining 427 participants of the SYS, was genotyped with the HumanOmniExpress BeadChip (Illumina, San Diego, CA; *n* = 729,295 SNPs) at the Genome Analysis Centre of Helmholtz Zentrum München (Munich, Germany). In both subsamples, SNPs with call rate <95% and minor allele frequency <0.01 and SNPs that were not in Hardy-Weinberg equilibrium (*P* < 1 × 10^−6^) were excluded. After this genotype quality control, 542,345 markers on the first SNP chip and 644,283 markers on the second chip were available for analysis.

**Figure 3:**
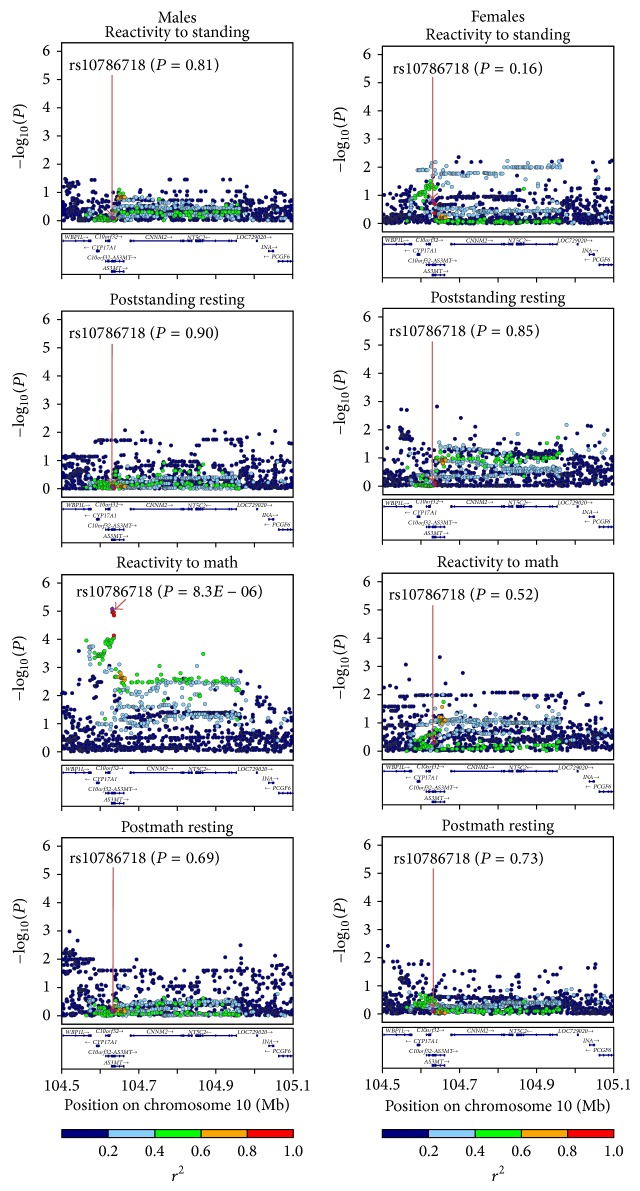
*CYP17A1* locus and SBP in response to physical and mental challenges and at rest. Individual points indicate −log_10_ (*P* values) for associations of SNPs within the* CYP17A1* locus and studied SBP phenotypes. Plots on the left and right show the results in males and females, respectively. Data were adjusted for age, height, and, when appropriate (SBP reactivity to standing and mental stress), initial SBP. The SNP, rs10786718, demonstrating the strongest association with SBP reactivity to math stress in males is indicated in purple and is the index SNP in all plots. The correlation (*r* ^2^) between this index SNP, rs10786718, and each of the other tested SNPs in the region is shown in red (1 ≥ *r* ^2^ ≥ 0.8), orange (0.8 > *r* ^2^ ≥ 0.6), green (0.6 > *r* ^2^ ≥ 0.4), light blue (0.4 > *r* ^2^ ≥ 0.2), or dark blue (0.2 > *r* ^2^ ≥ 0) colors. Gene positions are indicated at the bottom. The LD was calculated based on the 1,000 Genomes Project (EUR reference panel, March 2012 version); the chromosome positions are based on human genome hg19.

Genotype imputation was used to equate the set of SNPs of the adolescents genotyped on each platform and to increase the SNP density. Haplotype phasing was performed with SHAPEIT [33] using an overlapping subset of 313,653 post-quality-control SNPs that were present on both genotyping platforms and the 1,000 Genomes SNPs in European reference panel (Phase 1, Release 3). Imputation was conducted on the phased data with IMPUTE2 [34]. Markers with low imputation quality (information score < 0.5) or low minor allele frequency (<0.01) were removed. After this imputation quality control, a total of 1,392 typed and imputed SNPs were available for a segment of human chromosome 10 that covered the entire previously reported* CYP17A1* locus of adult SBP, DBP, and hypertension (~400 kb from* CYP17A1* to* NT5C2* [17]) and its flanking sequences (90 kb upstream of* CYP17A1* and 147 kb downstream of* NT5C2*, Figure 3 and Figure S4; see Supplementary Material available online at http://dx.doi.org/10.1155/2015/734586).

### 2.4. Statistical Analyses

Genotype-phenotype association analyses were conducted with Merlin (version 1.1.2) [35, 36] under an additive model. They involved the above-selected 1,392 SNPs covering the previously reported* CYP17A1* locus [17] and the following SBP and DBP phenotypes: (1) BP reactivity to math, (2) BP reactivity to standing, (3) poststanding resting BP, and (4) postmath resting BP. With Merlin, a simple regression model is fitted to each trait, and a variance component approach is used to account for correlation between observed phenotypes within sibships. Analyses were done in males and females separately, with age, height, and, when appropriate, initial BP included as covariates. In supplementary analyses, body weight was added as an additional covariate to control for potentially confounding effects of obesity. BP values outside the mean ± three standard deviations were excluded prior to these analyses (*n* = 974–992 per variable). As we used a candidate-gene approach, *P* < 0.05 was deemed significant for one test. However, since we tested 4 blood pressure phenotypes in 2 sexes, our results were considered significant at *P* < 0.05/8 ≤ 6.3 × 10^−3^ when Bonferroni's correction is applied. LocusZoom [37] was used to view the results of the above-described genotype-phenotype association analyses as plots showing both the magnitude of association for each SNP (1,392 SNPs within the* CYP17A1* locus) and the pairwise linkage disequilibrium (LD) pattern with the most strongly associated SNP (Figure 3). These plots were generated based on human genome hg19, and the LD was calculated based on the 1,000 Genomes Project (EUR reference panel, March 2012 version).

## 3. Results

The genotype-phenotype association analyses were carried out while adjusting for age, height, and initial BP when relevant. These analyses showed that, for both SBP and DBP, the* CYP17A1* locus was associated with enhanced BP reactivity to the mental but not physical challenge, and this was observed in males but not in females (Figure 3 and Table 2). The most significantly associated SNP was rs10786718 (Figure 3). SBP increase in response to math stress was higher in major versus minor allele homozygotes by 7.6 mm Hg (*P* = 8.3 × 10^−6^) in males. Likewise, DBP increase in response to math stress was higher in major versus minor allele homozygotes by 4.8 mm Hg (*P* = 1.1 × 10^−5^) in males (Figure S1). Neither poststanding nor postmath resting BPs were associated with the* CYP17A1* locus in either sex (Figure 3 and Table 2). All these results remained virtually unchanged when additionally adjusted for body weight (Figures S2 and S3), indicating that the* CYP17A1* locus impacts BP independently of obesity. Similar to the previous studies, the* CYP17A1* locus showed a long-range LD pattern, spreading over a region of ~400 kb [17]. In the present study, however, this locus contained a subregion of highly correlated SNPs that demonstrated a stronger association with the BP phenotype and was closer to* CYP17A1* (Figure 3); this observation provides further support for the possibility that* CYP17A1* may be the causal gene within this BP locus.

## 4. Discussion

The results of the current study show that a well-established gene locus of hypertension in adults [16–22],* CYP17A1*, may play a role in increasing BP reactivity to stress during adolescence. This effect, however, may be sex-specific and limited to mental stressors.

*CYP17A1* has been identified as a gene locus of hypertension and resting blood pressure in several large-scale genome-wide and replication studies of adult European and Asian populations [16–20]. It encodes a key enzyme in the steroidogenic pathway that may influence BP through its effects on the production of mineralocorticoids, glucocorticoids, and sex hormones. The enzyme has both 17*α*-hydroxylase and 17,20-lyase activities that contribute to steroidogenesis in the adrenal gland and sex gonads [24]. During that process, pregnenolone derived from cholesterol is processed to (a) mineralocorticoids in the adrenal zona glomerulosa (neither enzyme activity present), (b) glucocorticoids in the adrenal zona fasciculata (17*α*-hydroxylase activity present), or (c) sex steroids in the adrenal zona reticularis and gonads (both enzyme activities present) [24]. Each of these steroid hormones plays a role in the regulation of BP. (a) Mineralocorticoids (e.g., aldosterone) can increase BP by augmenting renal sodium and water reabsorption and thus circulating blood volume [23]. (b) Glucocorticoids (e.g., cortisol) can increase BP by enhancing the vasoconstrictive effects of catecholamines, as well as by having some mineralocorticoid activity described above [23]. (c) Sex steroids impact BP mostly indirectly, via their influences on the autonomic and renin-angiotensin-aldosterone systems, endothelial function, and oxidative stress; in general, androgens are considered to be BP-increasing, while estrogens are thought to be BP-decreasing [12, 38]. Importantly, some of the above effects of steroid hormones underlie slower BP responses (e.g., blood volume regulation) whereas other effects mediate faster BP responses (e.g., the enhancement of catecholamine-driven vasoconstriction). The latter ones are more likely to be relevant to the present study of acute BP reactivity.

The regulation of BP reactivity to mental stress involves the coordinated action of the hypothalamic-pituitary-adrenal (HPA) and autonomic nervous systems, whereas the regulation of BP reactivity to simple standing up engages only the autonomic system (baroreflex) [39, 40]. Our results show that the* CYP17A1* locus is associated with BP reactivity to mental stress but not to active standing up. This observation is consistent with mental stress (but not active standing up) involving the activation of the HPA axis and* CYP17A1* regulating the production of its final effector, cortisol [23]. Further, our results suggest that* CYP17A1* is involved in the regulation of BP reactivity to mental stress mainly in males. SBP increase in response to math stress was higher in major versus minor allele homozygotes by 6.3 mm Hg (*P* = 8.3 × 10^−6^) but only by 1.6 mm Hg (*P* = 0.52) in females. Although* CYP17A1* increases the production of both male and female sex hormones, androgens are expected to enhance BP reactivity, whereas estrogens are predicted to suppress BP reactivity [12, 38]. As such, sex hormones, through their opposing modulatory effects, may lead to the development of the observed sex dimorphism in the genotype-phenotype association. Taken together,* CYP17A1* may contribute to the regulation of BP reactivity to mental stress through its parallel effects on the production of cortisol and sex hormones.

Finally, our study did not observe significant association between* CYP17A1* and resting BP in either males or females. It is likely that, at this stage, the effect of the gene is detectable on BP reactivity but not yet on resting BP. Previous research suggests that enhanced BP reactivity increases risk for future hypertension [6–8]. Chronically exaggerated BP responses increase repeatedly pressure load on the vessels, heart, and kidneys, leading to their structural and functional adaptations, which in turn may increase resting BP and promote the development of hypertension over time. Given that* CYP17A1* may enhance BP reactivity to mental stress, it may be an early genetic marker of hypertension risk. Substantial overlap exists between genes influencing clinic BP and BP measured in an experimental setting. It has been estimated that up to 81% of the heritability of clinic SBP and 71% of the heritability of clinic DBP were attributed to genes that also influenced stress BP during a video game challenge and social stress interview [41]. Although this locus is a well-established locus of hypertension in adults [16–22], further studies in adolescents and young adults are needed to elucidate its potential age and sex dependence and phenotypic transition from enhanced reactivity to elevated resting blood pressure.

Our results remained virtually unchanged when adjusted, or not adjusted, for body weight (Figures S2 and S3), indicating that the* CYP17A1* locus impacts BP independently of obesity. As such, the locus may be relevant obesity-dissociated hypertension [42]. Although obesity is a leading risk factor of hypertension, obesity and hypertension do not have 1 : 1 correspondence; only about 50% of obese individuals are hypertensive and about 30% of hypertensive individuals are not obese [43].

The* CYP17A1* locus was discovered as a relatively wide locus, including several genes [17]. It was named the* CYP17A1* locus, as* CYP17A1* was considered the best physiological candidate within the association locus. Nevertheless, other genes within the identified locus cannot be entirely excluded. The two genes closest to* CYP17A1* are* C10orf32*, which encodes “chromosome 10 open reading frame 32” (a protein of unknown function), and* AS3MT,* which encodes an enzyme that catalyzes the transfer of a methyl group from S-adenosyl-L-methionine to trivalent arsenical and may play a role in arsenic metabolism [44]. However, the role of these two genes in blood pressure regulation is not clear.

In conclusion, our results suggest that, in adolescent males but not females,* CYP17A1* enhances BP reactivity to mental stress. Whether this effect contributes to the higher prevalence of hypertension in males than females later in life remains to be determined.

## Supplementary Material

Supplementary Figure S1: *CYP17A1* locus and DBP in response to physical and mental challenges and at rest.
Supplementary Figure S2: *CYP17A1* locus and SBP in response to physical and mental challenges and at rest (additionally adjusted for adiposity).
Supplementary Figure S3: *CYP17A1* locus and DBP in response to physical and mental challenges and at rest (additionally adjusted for adiposity).
Supplementary Figure S4: The *CYP17A1* locus reported previously in *Nature Genetics* (left) and in our present study (right).

## Figures and Tables

**Table 1:** Basic characteristics and studied BP phenotypes.

	Males	Females	Estimated difference (95% CI)	*P* value
(A) Basic characteristics^*^				
Age (years)	14.4 ± 1.8	14.6 ± 1.9	0.2 (−0.1–0.4)	0.15
Height (cm)	166.8 ± 10.7	159.6 ± 6.7	7.6 (6.7–8.6)	<0.0001
Body weight (kg)	61.3 ± 16.9	56.0 ± 12.6	0.9 (−0.7–2.5)	0.29
BMI (kg/m^2^)	21.8 ± 4.6	21.9 ± 4.4	0.02 (−0.5–0.6)	0.94
(B) SBP, mm Hg^†^				
Prestanding	118.7 ± 12.8	120.1 ± 11.3	1.4 (−0.2–3.1)	0.09
Standing	129.6 ± 15.4	129.0 ± 13.5	0.6 (−1.4–2.6)	0.53
Reactivity to standing	11.1 ± 9.8	8.8 ± 9.2	2.3 (1.0–3.6)	0.0007
Premath stress	122.6 ± 14.2	119.7 ± 13.0	2.9 (1.0–4.8)	0.002
Math stress	140.4 ± 16.3	136.2 ± 14.6	4.3 (2.2–6.4)	<0.0001
Reactivity to math stress	18.2 ± 10.6	16.7 ± 10.6	1.5 (0–3.0)	0.05
Poststanding resting^c^	123.2 ± 13.6	120.0 ± 11.8	3.1 (1.4–4.9)	0.0004
Postmath resting^c^	127.3 ± 13.1	124.8 ± 12.5	2.6 (0.8–4.3)	0.004
(C) DBP, mm Hg^†^				
Prestanding	70.3 ± 8.1	68.4 ± 7.4	1.9 (0.8–3.0)	0.0006
Standing	80.4 ± 11.1	78.8 ± 10.1	1.6 (0.1–3.1)	0.03
Reactivity to standing	10.4 ± 5.9	10.2 ± 5.8	0.2 (−0.7–1.0)	0.69
Premath stress	78.7 ± 9.6	76.1 ± 9.2	2.6 (1.3–3.9)	0.0001
Math stress	89.0 ± 11.4	86.6 ± 10.6	2.4 (0.8–3.9)	0.002
Reactivity to math stress	10.4 ± 5.8	10.6 ± 6.0	0.2 (−0.7–1.0)	0.67
Poststanding resting^c^	78.9 ± 9.6	76.1 ± 9.0	2.8 (1.5–4.1)	<0.0001
Postmath resting^c^	81.9 ± 9.7	79.8 ± 8.9	2.2 (0.9–3.5)	0.001

^*^Nonadjusted means ± standard deviations are shown. The differences between males and females are given, adjusted for age and, when appropriate, height.

^†^Means adjusted for age and height ± standard deviations are shown. The differences between males and females are given, adjusted for age and height.

^c^Resting SBP/DBP is a 5-minute average of SBP/DBP measured while seated after 5 minutes at rest.

CI = confidence interval.

**Table 2:** Associations of the* CYP17A1* locus (rs10786718^#^) and studied BP phenotypes.

	Males					Females				
	Beta (SE)	AA	GA	GG	*P* value	Beta (SE)	GG	GA	AA	*P* value
		Mean (SD)	Mean (SD)	Mean (SD)			Mean (SD)	Mean (SD)	Mean (SD)	
(A) SBP										
Reactivity to standing	−0.17 (0.70)	8.5 (1.5)	11.8 (0.8)	10.1 (0.6)	0.81	0.92 (0.66)	9.5 (0.5)	9.4 (0.7)	7.6 (1.4)	0.16
Poststanding resting^*^	0.12 (0.98)	120.9 (2.0)	125.7 (1.0)	123.5 (0.8)	0.90	−0.17 (0.85)	118.7 (0.7)	119.8 (0.9)	117.9 (1.9)	0.85
Reactivity to math	3.40 (0.76)	12.4 (1.6)	17.3 (0.8)	20.0 (0.6)	8.3*E* − 06	0.48 (0.75)	16.5 (0.6)	16.8 (0.8)	14.4 (1.6)	0.52
Postmath resting^*^	0.38 (0.95)	125.6 (2.0)	129.1 (1.0)	127.8 (0.8)	0.69	0.31 (0.90)	124.0 (0.7)	123.9 (1.0)	123.6 (2.0)	0.73
(B) DBP										
Reactivity to standing^*^	0.09 (0.42)	9.2 (0.9)	10.5 (0.5)	10.1 (0.4)	0.83	0.23 (0.42)	10.4 (0.4)	10.5 (0.5)	9.3 (0.9)	0.58
Poststanding resting	−0.07 (0.68)	77.3 (1.5)	80.6 (0.7)	78.9 (0.6)	0.92	0.44 (0.65)	75.3 (0.5)	76.5 (0.7)	72.5 (1.4)	0.50
Reactivity to math^*^	1.85 (0.42)	6.6 (0.9)	10.5 (0.4)	11.4 (0.4)	1.1*E* − 05	0.24 (0.42)	10.5 (0.4)	10.5 (0.5)	9.4 (0.9)	0.57
Postmath resting	0.03 (0.69)	80.6 (1.5)	83.0 (0.7)	81.9 (0.6)	0.97	0.30 (0.66)	79.3 (0.5)	79.7 (0.7)	77.8 (1.5)	0.65

Beta (SE = standard error) is the effect size on BP in mm Hg, per 1 copy of the index allele (G) based on an additive model adjusted for age and height.

^*^Model was additionally adjusted for initial BP.

^#^Minor allele (A) frequencies in males and females are 0.27 and 0.26, respectively.

## Perspectives

Adolescence is a period of human development when adult physiology develops and initial stages of cardiovascular disorders, including hypertension, may emerge. Our hope is that it is also a period when disease trajectories can still be altered and full-blown diseases (such as hypertension) prevented. Early vulnerability genes may represent useful tools in this effort.
