# Supplementary material for: CYP17A1 and Blood Pressure Reactivity to Stress in Adolescence
Source: Int J Hypertens. 2015 Jan 26;2015:734586. doi: 10.1155/2015/734586 (PMC4321855; doi:10.1155/2015/734586)
Supplement: Supplementary file 1 — Supplementary Figure S1: CYP17A1 locus and DBP in response to physical and mental challenges and at rest. Supplementary Figure S2: CYP17A1 locus and SBP in response to physical and mental challenges and at rest (additionally adjusted for adiposity). Supplementary Figure S3: CYP17A1 locus and DBP in response to physical and mental challenges and at rest (additionally adjusted for adiposity). Supplementary Figure S4: The CYP17A1 locus reported previously in Nature Genetics (left) and in our present study (right). [file 734586.f1.pdf]

SUPPLEMENTAL MATERIAL

Figure S1. *CYP17A1* locus and DBP in response to physical and mental challenges and at rest

Individual points indicate −log10(p-values) for associations of SNPs within the *CYP17A1* locus and studied DBP phenotypes. Plots on the top and bottom show the results in males and females, respectively. Data were adjusted for age, height and, when appropriate (DBP reactivity to standing and mental stress), initial DBP. The SNP, rs10786718, demonstrating the strongest association with SBP reactivity to math stress in males is indicated in purple and is the index SNP in all plots. The correlation (r^2) between this index SNP, rs10786718, and each of the other tested SNPs in the region is shown in red ($1 \geq r^2 \geq 0.8$), orange ($0.8 > r^2 \geq 0.6$), green ($0.6 > r^2 \geq 0.4$), light blue ($0.4 > r^2 \geq 0.2$), or dark blue ($0.2 > r^2 \geq 0$) colors. Gene positions are indicated at the bottom. The LD was calculated based on the 1,000 Genomes Project (EUR reference panel, March 2012 version); and the chromosome positions are based on Human Genome hg19.

Figure S2. *CYP17A1* locus and SBP in response to physical and mental challenges and at rest (additionally adjusted for adiposity)

Individual points indicate −log10(p-values) for associations of SNPs within the *CYP17A1* locus and studied SBP phenotypes. Plots on the top and bottom show the results in males and females, respectively. Data were adjusted for age, height, body weight and, when appropriate (SBP reactivity to standing and mental stress), initial SBP. The SNP, rs10786718, demonstrating the strongest association with SBP reactivity to math stress in males is indicated in purple is the index SNP in all plots. The correlation (r^2) between this index SNP, rs10786718, and each of the other tested SNPs in the region is shown in red ($1 \geq r^2 \geq 0.8$), orange ($0.8 > r^2 \geq 0.6$), green ($0.6 > r^2 \geq 0.4$), light blue ($0.4 > r^2 \geq 0.2$), or dark blue ($0.2 > r^2 \geq 0$) colors. Gene positions are indicated at the bottom. The LD was calculated based on the 1,000 Genomes Project (EUR reference panel, March 2012 version); and the chromosome positions are based on Human Genome hg19.

Figure S3. *CYP17A1* locus and DBP in response to physical and mental challenges and at rest (additionally adjusted for adiposity)

Individual points indicate −log10(p-values) for associations of SNPs within the *CYP17A1* locus and studied DBP phenotypes. Plots on the top and bottom show the results in males and females, respectively. Data were adjusted for age, height. Body weight and, when appropriate (DBP reactivity to standing and mental stress), initial DBP. The SNP, rs10786718, demonstrating the strongest association with DBP reactivity to math stress in males is indicated in purple is the index SNP in all plots. The correlation (r^2) between this index SNP, rs10786718, and each of the other tested SNPs in the region is shown in red ($1 \geq r^2 \geq 0.8$), orange ($0.8 > r^2 \geq 0.6$), green ($0.6 > r^2 \geq 0.4$), light blue ($0.4 > r^2 \geq 0.2$), or dark blue ($0.2 > r^2 \geq 0$) colors. Gene positions are indicated at the bottom. The LD was calculated based on the 1,000 Genomes Project (EUR reference panel, March 2012 version); and the chromosome positions are based on Human Genome hg19.

Figure S4. The *CYP17A1* locus reported previously in *Nature Genetics* (left) and in our present study (right).

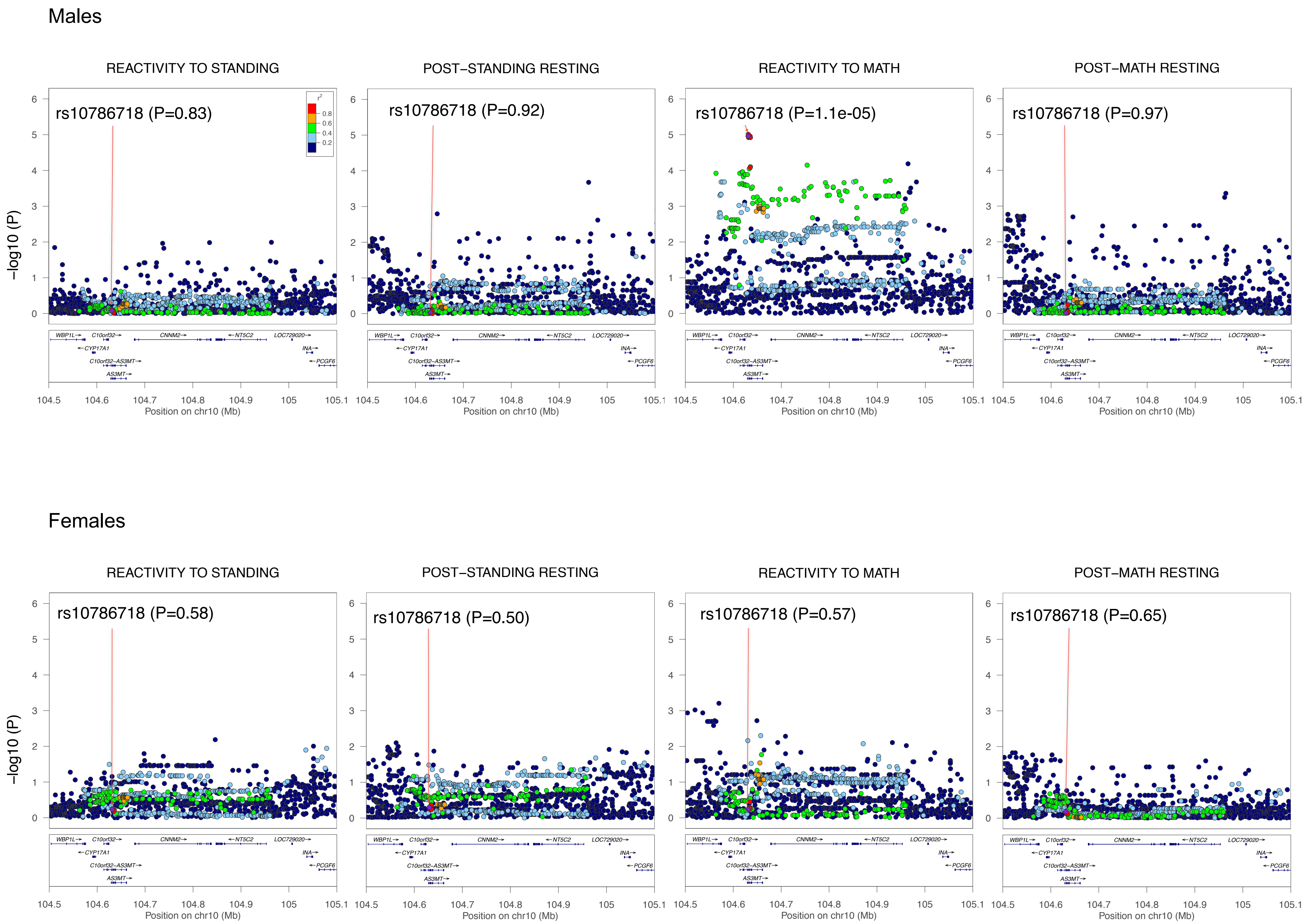

Figure S1. CYP17A1 locus and DBP in response to physical and mental challenges and at rest

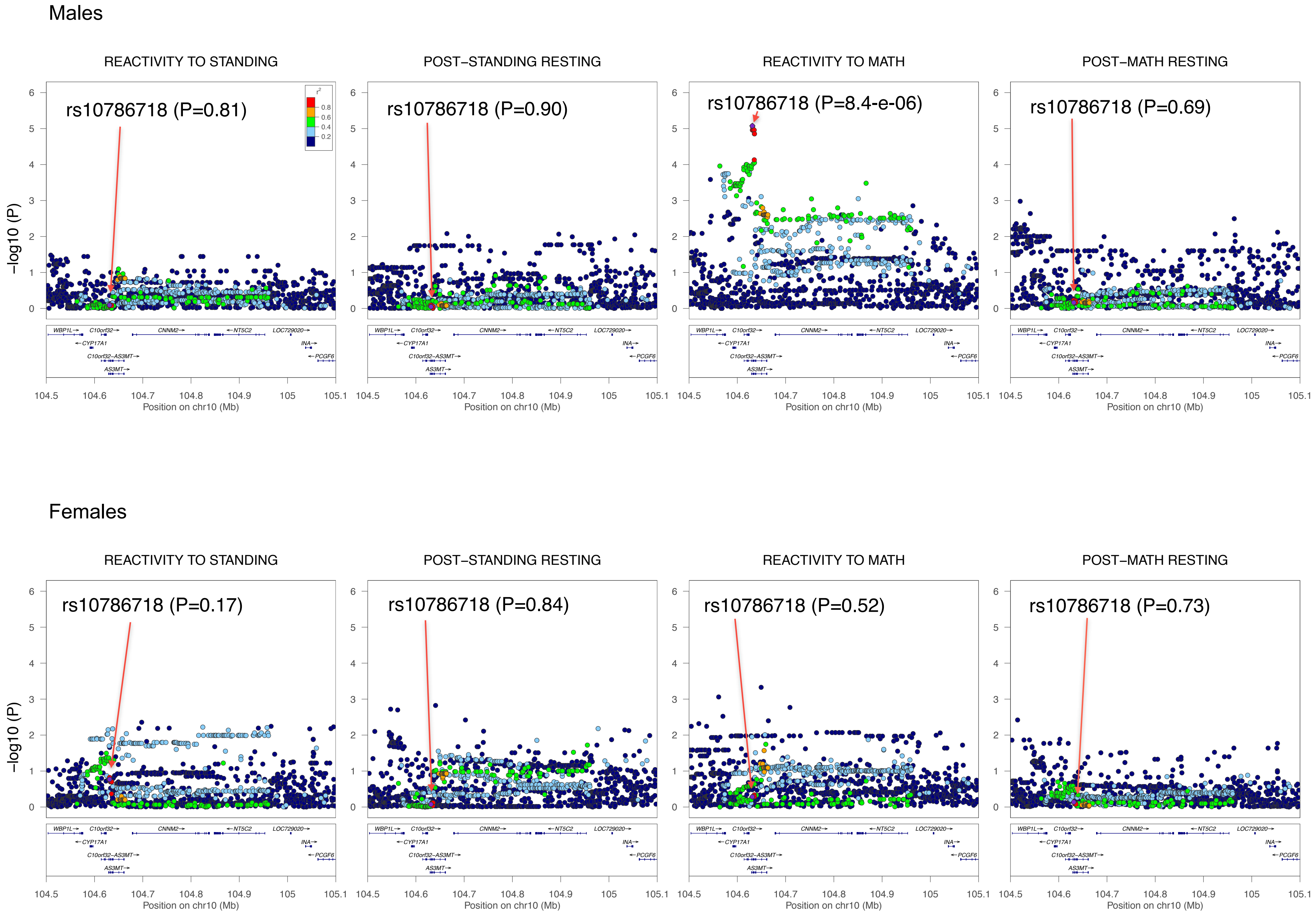

Figure S2. CYP17A1 locus and SBP in response to physical and mental challenges and at rest (additionally adjusted for adiposity)

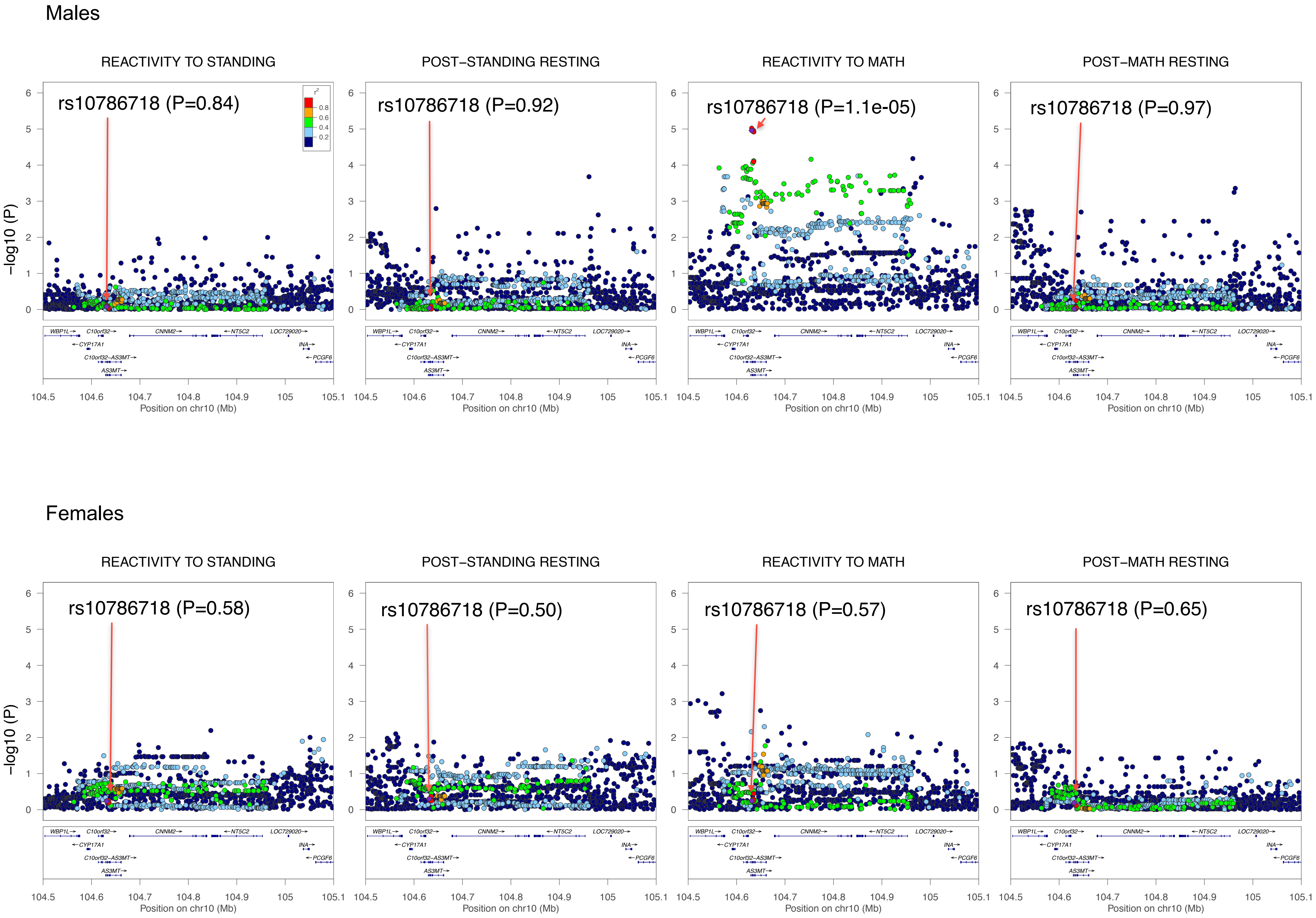

Figure S3. CYP17A1 locus and DBP in response to physical and mental challenges and at rest (additionally adjusted for adiposity)

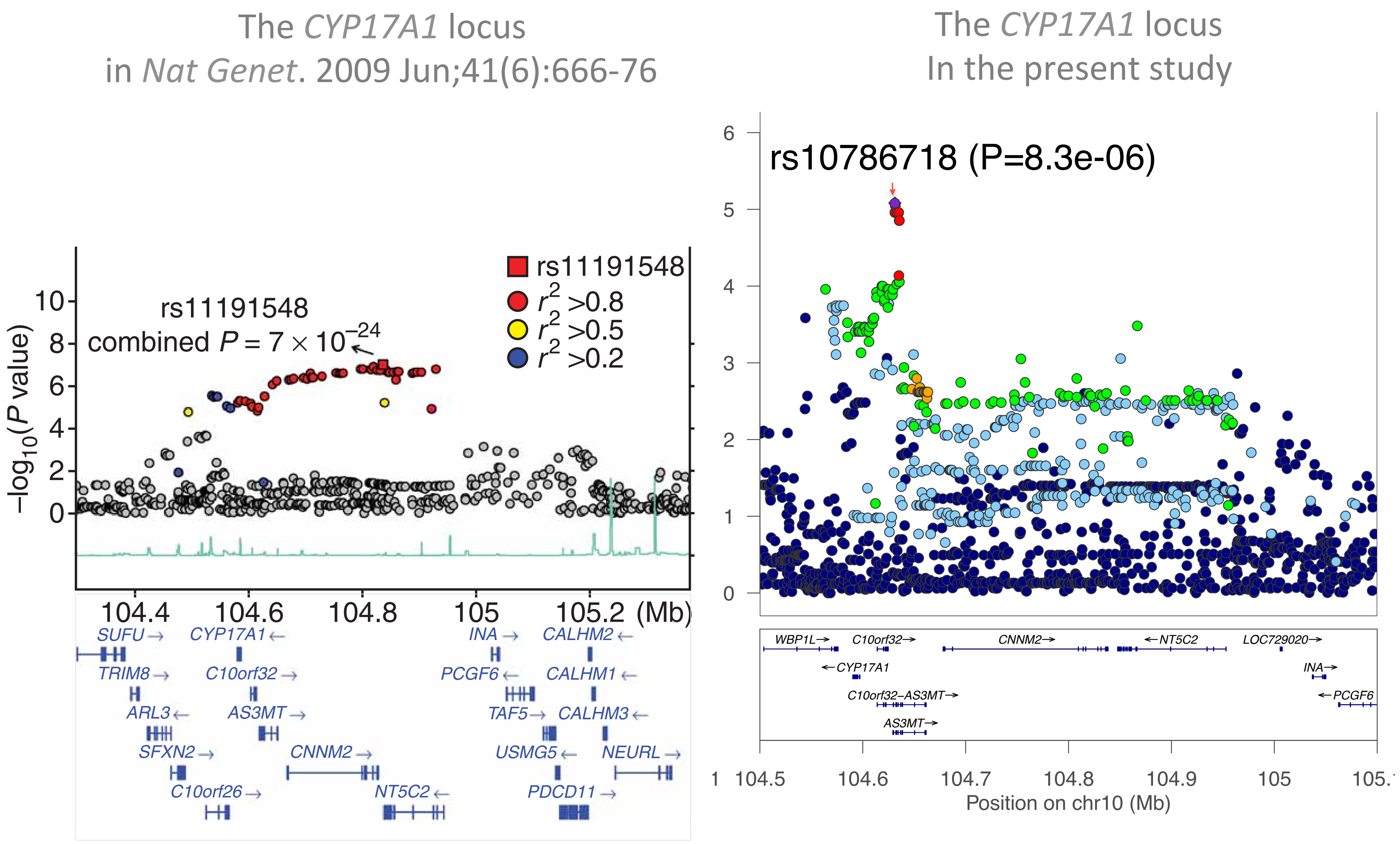

Figure S4. The *CYP17A1* locus reported previously in *Nature Genetics* (left) and in our present study (right).
